# Acute Myocardial Infarction following a possible direct intravenous bite of Russell’s viper (*Daboia russelli*)

**DOI:** 10.1186/1756-0500-5-500

**Published:** 2012-09-12

**Authors:** Anjana Silva, Senaka Pilapitiya, Sisira Siribaddana

**Affiliations:** 1 Department of Parasitology, Faculty of Medicine and Allied Sciences, Rajarata University of Sri Lanka, Anuradhapura, Sri Lanka; 2 Department of Medicine, Faculty of Medicine and Allied Sciences, Rajarata University of Sri Lanka, Anuradhapura, Sri Lanka; 3 Institute of Research and Development, Battaramulla, Sri Lanka

**Keywords:** Myocardial infarction, Russell’s viper, Intravenous, Sri Lanka

## Abstract

**Background:**

Russell’s viper (*Daboia russelli*) bites lead to high morbidity and mortality in South Asia. Although variety of clinical manifestations is reported in viper bite victims, myocardial ischemic events are rare.

**Case presentation:**

We report a unique case of inferior wall ST elevation myocardial infarction due to a Russell’s viper bite over a vein with possible direct intravenous envenoming, in a young male with no past history or family history suggestive of ischemic cardiac disease, from Sri Lanka. In addition, the possible mechanisms of myocardial ischemia in snake bite victims are also briefly discussed.

**Conclusion:**

Importance of the awareness of physicians on the rare, yet fatal manifestations of snake envenoming is highlighted.

## Background

Envenoming by Russell’s viper (*Daboia russelli*) is common in Sri Lanka, particularly in North Central Province where it leads to highest number of bites than any other snake [1], causing frequent systemic envenoming [2,3]. Local swelling (92%), local necrosis (8.9%), coagulopathy (77%), neurotoxicity (78%), nephrotoxicity (18%), cardiac effects (3-12%) and myotoxicity (14%) is known to be associated with envenoming by Russell’s vipers [2]. The available antivenom for this snake (Indian polyvalent antivenom), is known to be less effective in treating victims bitten by Sri Lankan Russell’s viper [3].

Ischemic cardiac events [2], arrhythmias [4] and cardiac tamponade [5] rarely occur after Russell’s viper bites. Although very rare, myocardial infarction following Russell’s viper bite has been previously reported from Sri Lanka [6]. We report rapid development of inferior wall ST elevation myocardial infarction after Russell’s viper bite, probably due to direct intravenous venom injection.

## Case presentation

**Figure 1:**
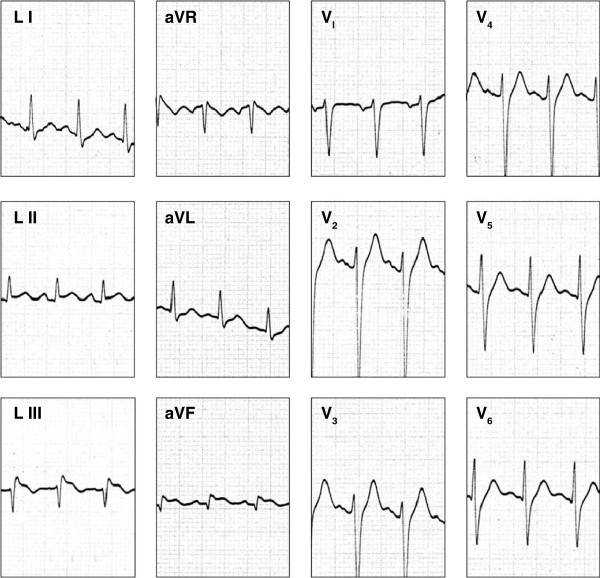
** Twelve- lead Echocardiogram of the victim, taken 2 hours after the viper bite: note the ST elevations in L**_**III**_** and aVF leads.**

A 33 year old male was admitted to the Emergency Treatment Unit of the Teaching Hospital, Anuradhapura, Sri Lanka, at 07:15 p.m on 09^th^ April 2012, following a Russell’s viper bite to his right ankle. The bite had taken place at 06:30 p.m on the same day when he was returning home after fishing, on a foot path from an inland reservoir. The patient identified the offending snake and noticed blood gushing out from the bite site. The assailant snake was not collected. He developed tightness of the chest and syncope within few minutes after the bite. On admission, the patient was unconscious without spontaneous breathing or heart rhythm. Following cardio-pulmonary resuscitation at the Emergency Treatment Unit, he had sinus rhythm with BP of 150/110 mmHg and pulse rate of 120 min^-1^and was immediately intubated and transferred to Medical Intensive Care Unit. He was ventilated with intermittent positive pressure ventilation. His right ankle was swollen with two fang marks over a dilated vein anterior to the medial malleolus. With the blood pressure picking up following the resuscitation, profuse bleeding from the fang marks was observed. Bleeding settled following application of a tight dressing. His 12 lead ECG taken 2 hours after the bite (Figures 1) showed sinus tachycardia, ST elevations in L_III_ and aVF leads and depressed ST segments in aVL. The ECG changes were suggestive of inferior wall myocardial infarction. In addition, his Troponin I assay was found to be positive. The patient had no past history or family history of ischemic heart disease. His on-admission Whole Blood Clotting Time (WBCT) exceeded 20 minutes. The full blood count showed marked leukocytosis with a total white blood cell count of 27600/μl with 72% Neutrophils. An infusion of 20 vials of Indian Polyvalent Anti-venom Serum (Bharat Serums and Vaccines Ltd, India) in 200 ml of normal saline was infused along with intravenous injections of Hydrocortisone, Promethazine and Chlopheniramine. Intravenous Cefotaxime one gram eight hourly were given as a prophylactic antibiotic. The victim developed haematuria four hours following the bite and the repeat WBCT too exceeded 10 minutes. Hence, a repeat infusion of 20 vials of Anti-venom Serum was given. Repeat ECGs on day 1 and day 2 displayed the same changes seen in the 1^st^ ECG. Two dimensional echocardiogram, was normal but his blood pressure was persistently high in the range of 170/110 - 160/100 mmHg. It required intravenous Glyceryl Trinitrate 0.5 mg/hr infusion. Since day 1, WBCT was found to be normal and no haematuria was observed. His serum electrolytes were normal. However, his urine output decreased gradually and the blood urea was 125 mg/dl by the day 2. The victim never recovered from the state of unconsciousness and died following repeated cardiac arrests on 12.04.2012, 58 hours after the bite.

Teaching Hospital - Anuradhapura is located in the North Central Province, where highest number of Russell’s viper bites is recorded from Sri Lanka [1]. Of all the snakebite admissions to this hospital, 48% are due to Russell’s viper bites [7]. In Sri Lanka, bites by these snakes are highest in months of March-April, and commonly occur at daytime on paddy fields and at dusk on footpaths and roads [2]. Fang marks are almost always associated with Russell’s viper bites and 77% of victims develop prolonged WBCT [2]. Although the assailant snake was not available for authentication, the victim identified the snake and told others accompanying him to the hospital, the geographical area, time and circumstance of the bite, prominent fang marks and prolonged WBCT all indicate to Russell’s viper bite.

Cardiac manifestations are relatively uncommon in snake bite victims, compared to more common systemic manifestations like coagulopathy, renal toxicity or neurotoxicity. Cardiac manifestations seem to be more rapid and life threatening than most of the other systemic manifestation of snake envenoming. Reported case of myocardial infarction had occurred within 1 hour following Russell’s viper bite, before other systemic manifestations [6].

Based on Troponin T levels and ECG of 13 victims of Sri Lankan Russell’s viper, Seneviratne et al. [8] opined that myocardial damage is not an important feature of Russell’s viper bites in Sri Lanka. Kularatne [2] observed ischemic EEG changes only in four of the 336 Russell’s viper bite victims from Sri Lanka. However in this case, ST elevations in L_III_ and aVF leads of the first ECG (Figures 1) and the changes in subsequent ECGs which were consistent with the first ECG suggests an inferior wall myocardial infarction, probably due to occlusion of the right coronary artery. The elevated Troponin I levels support the diagnosis of myocardial infarction, although this may be due to cardio- pulmonary resuscitation. The exact mechanism by which snake venom causes myocardial ischemia is unclear. However, hypovolemic shock, hypercoagulability , toxic myocarditis and coronary artery spasm have been proposed as possible mechanisms for myocardial infarction in snake bite victims, both humans and animals [9-13]. Hypovolemic shock and toxic myocarditis are unlikely in this patient as these lead to more extensive myocardial damage, and they take some time to develop in snake bite victims [12]. In addition, toxic myocarditis as the cause of the ECG changes with elevated cardiac enzymes is unlikely due to lack of associated arrhythmias.

Russell’s viper venom contains components like factor V and Factor X activators, which create high procoagulant potential within the bite victim [14]. Compared to small animals, this however, generally does not lead to thrombosis in humans because in usual snake bites venom injection is subcutaneous or intramuscular and the venom dose is small relative to the body weight [15]. Therefore, rather than thrombosis, this give rise to venom induced consumption coagulopathy in humans [16]. However, evidence for thrombosis in coronary, cerebral and pulmonary vasculature has been observed following viper bites in humans [11,13,17-20] and a possibility in this patient.

In most snake bites, the venom is being injected via intramuscular or subcutaneous route [14]. However, intravenous route provides more rapid and efficient envenoming. Hence the ‘usual’ clinical picture may vary when the route of venom injection is intravenous. In this victim, gush of blood from the bite site, fang marks over a dilated vein and rapid bleeding through the fang marks with the rise of blood pressure following resuscitation indicate a direct intravenous venom injection. In addition, development of syncope within few minutes and the cardiac arrest, 45 minutes after the bite may also indicate rapid and severe envenoming, which could well be due to intravenous venom injection. Therefore, a high dose of venom entered via intravenous route leading to coronary thrombosis resulting in rapid development of myocardial infarction in this patient is a possibility.

Coronary artery spasm is also likely in cases of ischemic cardiac symptoms following the snake bite [12], Presence of normal coronary arteries in post myocardial infarction coronary angiograms of the viper bite induced myocardial infarction patients [9,21] suggest coronary artery spasm as a possible cause of myocardial ischemia. Similar ST elevation myocardial infarctions have been observed in viper bite victims who had developed coronary artery spasm following Kounis syndrome [22], which is a concurrence of acute coronary syndromes associated with mast cell activation including allergic and anaphylactic insults [23]. Clinically, we did not observe any evidence of above events in the victim on admission or following the recovery form the initial cardiac arrest, hence Kounis syndrome is less likely to have occurred in our patient. Hyperkalemia and cardiac arrest following Russell’s viper bites had been reported from Sri Lanka in two deaths [2]. However, the serum potassium levels of our patient remained within normal range throughout, thus this is a remote possibility.

However, since the post mortem was not conducted in this patient, it is not possible to draw any firm conclusions on the aetio-pathogenesis of the myocardial infarction.

Neutrophil leukocytosis has been commonly observed among viper bite victims [3,24], as similarly observed in this victim who had an on-admission count of 27.6 X 10^3^ μl^-1^. Although a common phenomenon, extensive literature search failed to find studies investigating mechanism of leukocytosis following snake envenoming.

## Conclusions

This unique case provides evidences for rapid development of ST elevation myocardial infarction following a possible intravenous envenoming by a Russell’s viper bite. Pathophysiology of myocardial ischemia in snakebites needs to be explored through careful documentation of clinical condition and thorough investigation of the snakebite victims.

## Consent

Since the patient was unconscious throughout, the written informed consent was obtained from the spouse of the patient for publication of this case report and accompanying figure. A copy of the written consent is available for review by the Editor-in-Chief of this journal.

## Abbreviations

ECG: Electrocardiogram; WBCT: Whole blood clotting time.

## Competing interests

The authors declare that they have no competing interest.

## Authors’ contributions

AS drafted the first manuscript, SP and SS managed the patient and improved the manuscript, all authors read and approved the final manuscript.
